# The impact of triglyceride-glucose index on ischemic stroke: a systematic review and meta-analysis

**DOI:** 10.1186/s12933-022-01732-0

**Published:** 2023-01-06

**Authors:** Ying Yang, Xiangting Huang, Yuge Wang, Lin Leng, Jiapei Xu, Lei Feng, Shixie Jiang, Jiang Wang, Yanrong Yang, Gaofeng Pan, Bing Jiang, Yan Wang, Lan Chen

**Affiliations:** 1 Geriatric Diseases Institute of Chengdu/Cancer Prevention and Treatment Institute of Chengdu, Department of Neurology, Chengdu Fifth People’s Hospital, (The Second Clinical Medical College, Affiliated Fifth People’s Hospital of Chengdu University of Traditional Chinese Medicine), Chengdu, China; 2 School of Computer Science and Technology, Chongqing University of Posts and Telecommunications, Chongqing, China; 3 Department of Medicine, Jinggangshan University, Ji’an, Jiangxi China; 4 The Centre of Gerontology and Geriatrics, West China Hospital, Sichuan University, Chengdu, China; 5 Department of Nephrology, Fifth People’s Hospital of Chengdu, Chengdu, China; 6 Department of Psychiatry and Behavioral Sciences, Stanford University School of Medicine, Stanford, CA USA; 7 Department of Neurology, Affiliated Hospital of Jinggangshan University, JingGangshan University Ji’an, Jiangxi province, 343000 China cljgsu@163.com,

**Keywords:** The triglyceride and glucose (TyG) index, Stroke, Prevalence, Adverse outcomes

## Abstract

**Background:**

Strokes significantly impair quality of life and incur high economic and societal burdens. The triglyceride and glucose (TyG) index is a biochemical marker of insulin resistance (IR) and may have important value in the prediction of strokes, especially ischemic stroke (IS). Our study aims to investigate the relationship between TyG index and IS and ascertain whether TyG index is independently associated with IS adverse outcomes.

**Methods:**

The Cochrane, Embase, Medline, Web of Science, PubMed, and other relevant English databases and related websites were systematically searched for articles on ‘‘TyG index’’ and "stroke" published from inception to April 4, 2022. We reviewed the available literature on the TyG index and its relation to predicting IS occurrence in the general population and adverse clinical outcomes. We calculated odds ratios (OR) of TyG index and its predictability of IS occurrence and adverse outcomes. Statistical analyses were performed using the Meta Package in STATA, version 12.0.

**Results:**

A total of 18 studies and 592,635 patients were included in our analysis. The pooled effect values of all stroke types showed that higher TyG index was associated with increased the risk of IS in the general population (OR 1.37; 95% CI 1.22–1.54) in a total sample of 554,334 cases with a high level of heterogeneity (P = 0.000, *I*^*2*^ = 74.10%). In addition, compared to IS patients with a lower TyG index, IS patients with a higher TyG index was associated with higher risk of stroke recurrence (OR: 1.50; 95% CI 1.19–1.89) and increased risk of mortality (OR 1.40 95% CI 1.14–1.71). No correlation was found in the effect value combinations of poor functional outcomes (OR 1.12; 95% CI 0.88–1.43) and neurological worsening (OR: 1.76; 95% CI 0.79–3.95) in a total sample of 38,301 cases with a high level of heterogeneity (P = 0.000; *I*^*2*^ = 77.20%).

**Conclusions:**

TyG index has potential value in optimizing risk stratification for IS in the general population. Furthermore, there is a significant association between high TyG index and many adverse outcomes of stroke, especially stroke recurrence and high mortality. Future studies should focus on multi-center and multi-regional designs in order to further explore the relationship between IS and TyG index.

**Supplementary Information:**

The online version contains supplementary material available at 10.1186/s12933-022-01732-0.

## Introduction

A stroke is an acute neurologic condition that occurs due to a disruption of cerebral perfusion, resulting in focal or global neurological impairment [1]. Strokes can be broadly classified into ischemic strokes (IS) and hemorrhagic strokes (HS). Approximately 84.4% of strokes are ischemic in origin [1]. Annually, over 13.7 million strokes occur globally and cause 5.5 million deaths per year as well, with a predilection for the elderly population, though increasing prevalence is being reported in younger adults [2, 3]. As stroke causes death, dementia, and disability worldwide, this common condition decreases quality of life and incurs high economic and societal burdens [4–6]. Despite the improvement of strategies and techniques towards the management of stroke patients in recent years, recurrence of strokes continue to account for nearly 30% of all strokes and this high rate likely represents unsuccessful secondary prevention [4, 7]. Researchers have recognized that identifying stroke-prone individuals and targeting them effectively remains an important part of stroke management; however, this is not an easy task [4]. Frans Kauwa demonstrated that predictors assessed with magnetic resonance imaging (MRI) including multiple ischemic changes and isolated cortical lesions may have potential, but computed tomography (CT) or ultrasound are not reasonable choices. Unfortunately, overuse of MRI to predict strokes was not found to be feasible, thus portraying the incomplete utility of neuroimaging in this endeavor [8]. There are several validated risk factors as a target for IS prevention, such as hypertension, diabetes mellitus, hyperlipidemia, hypercoagulable states, current smoking, atrial fibrillation (AF), and premature ventricular complexes (PVC). However, these risk factors fail to explain all cases of stroke and lack uniform applicability [4, 9].

The triglyceride-glucose (TyG) index, is a biochemical marker of insulin resistance (IR), and can be calculated as ln (fasting triglycerides (mg/dl)×fasting blood glucose (mg/dl)/2) [10, 11]. IR is known to be a key mediator of the pathogenesis of type 2 diabetes, and thus elevated stroke risk. Hyperinsulinemic-euglycemic clamp (HIEC) is considered the current gold standard to determine IR; however, HIEC is complicated and time-consuming with limited applicability for clinical practice on a large scale [11]. Therefore, the TyG index has been validated as a simple surrogate marker of IR and is cost-effective and reproducible [12]. After initial studies on its use in diabetes, many other publications now have been released which acknowledge its utility in other disorders. The TyG index has been associated with the severity of arterial stiffness, cardiovascular disease (CVD), and metabolic syndrome (MetS) (a cluster of metabolic abnormalities characterized by hypertension, dyslipidemia, obesity, and glucose dysregulation) [10, 12–15]. More interestingly, Jiao et al. also reported that a high TyG index was associated with a 1.64-fold risk of all-cause mortality and 1.36-fold risk of major adverse cardiac event in elderly acute coronary syndrome patients [10].

Stroke is one of the most significant causes of death and disability, with most of the burden in low-income and middle-income countries. Improving poststroke outcomes is an urgent issue worldwide. Several studies have investigated the association of TyG index with the risk of stroke, and its sub-types [16–18]. A prospective study of 54,098 participants demonstrated that patients with higher TyG index experienced a 1.30-fold increased risk of IS [19]. Huang et al. reported that long-term elevated TyG index in hypertensive patients was significantly associated with an increased risk of stroke, especially IS [20]. However, Zhao et al. demonstrated a high TyG index was not associated with nondiabetic patients with non-ST-elevation myocardial infarctions [21]. Additionally, the effect of TyG indices with studying prognosis among patients with IS also attracts great attention. A study of the 16,310 patients from the China National Stroke Registry II found that the TyG index was associated with 1.25-fold increased risk of all-cause mortality and 1.32-fold increased risk of stroke recurrence among patients with ischemic stroke [22]. This association was not found to be consistent in patients with stroke treated with intravenous thrombolysis [23].

Based on these seminal studies, there are strong implications and suggestions that the TyG index may be independently associated with stroke recurrence and clinical outcomes. Therefore, our present systematic review and meta-analysis aims to summarize and pool the current available data in order to analyze this relationship between the TyG index and IS.

## Method

The Meta-analysis was performed according to Preferred Reporting Items for Systematic Reviews and Meta-Analyses (PRISMA) guidelines [24]. The study protocol was registered on the International Platform of Registered Systematic Review and Meta-analysis Protocols (registration number: INPLASY2022110145).

### Search strategy

We searched the Cochrane Library, Embase, Medline, Web of Science, PubMed, and other relevant English databases and related websites from inception to 4 April 2022. A Google search was also conducted accordingly. We used the following appropriate Medical Subject Headings (Mesh) and free-text words to identify "triglyceride glucose index ‘‘and ’’stroke or cerebrovascular apoplexy or cerebrovascular stroke or cerebral stroke’’. The search terms and algorithm are detailed in Additional file 1: Table S1. There were no additional restrictions on time, country, or language, but ‘‘human only’’ was restricted for subjects. In addition, we manually checked the reference list of all the identified studies to search for other relevant articles.

### Study selection

The inclusion criteria of the meta-analysis were as follows: (1) studies investigating the association of TyG index and the risk of IS or investigating the TyG index level with clinical outcome among patients with IS; (2) longitudinal cohort studies or cross-sectional studies; (3) adult (age > 18 years) individuals of any sex or ethnicity. The exclusion criteria of the meta-analysis were as follows: (1) The study participants included only hemorrhagic stroke; (2) conference proceedings; (3) experimental, interventional studies, reviews, case reports; (4) studies with insufficient data; (4) non-English language studies. Two reviewers (YY and HXT) independently screened the literature based on pre-established inclusion and exclusion criteria to initially identify potentially relevant studies. Any difference in the included studies was solved by discussion with another author (WY).

### Data extraction

Data were extracted by two researchers (YY and HXT) independently by using the same Excel spreadsheets of related items formulated in advance, including the first author, the year of publication, country, the proportion of males, the mean age, study population, sample size, study design, study follow-up time, the definition of the TyG index, effect sizes for the association of TyG index with risk of IS or the effects of TyG index with prognosis among IS patients. After comparison of the extracted information, any differences were resolved by another author (WY).

### Assessment of quality

Appropriate scales were used for quality evaluation according to different study types. The Newcastle–Ottawa Quality Assessment Scale (NOS) was used for cohort studies (scores range from 0 to 9). Studies with a NOS score of  ≥ 7 were considered high quality. The Agency for Healthcare Research and Quality (AHRQ) scale used for analysis of any cross-sectional study. Given that the criteria contained 11 items, we considered studies with AHRQ scores ≥8 to be of high quality [25]. Different study types were assessed independently by two researchers (YY and FL) according to the appropriate scale. Similarly, any disagreements were resolved through discussion with another author (WY).

### Statistical analysis

STATA 12.0 software was used to perform this systematic evaluation and the statistical analysis for the meta-analysis. We conducted a meta-analysis to investigate the association of TyG index with risk of IS, and determine the effects of TyG index with prognosis among IS patients. For dichotomous data, the odds ratio (OR), and 95% confidence intervals (CI) were calculated. Due to the heterogeneity of the included studies, we used a random-effects model to pool the data for all outcomes. Chi-square tests and *I*^2^ statistics were used to determine heterogeneity. A significant difference in a study was considered when P < 0.05 and *I*^2^ > 50%. Forest plots were conducted for estimation of effect sizes based on 95% confidence intervals. In order to explore the source of heterogeneity, subgroup analyses were employed for included inter-study heterogeneity and meta-regression analyses tested for the source of covariate heterogeneity between subgroups. A sensitivity analysis was used to further examine the impact of individual studies on the overall outcomes and analyze whether the results were stable and reliable. The risk of bias was evaluated using the Begg's test and Egger’s test [26].

## Results

### Study selection

**Fig. 1:**
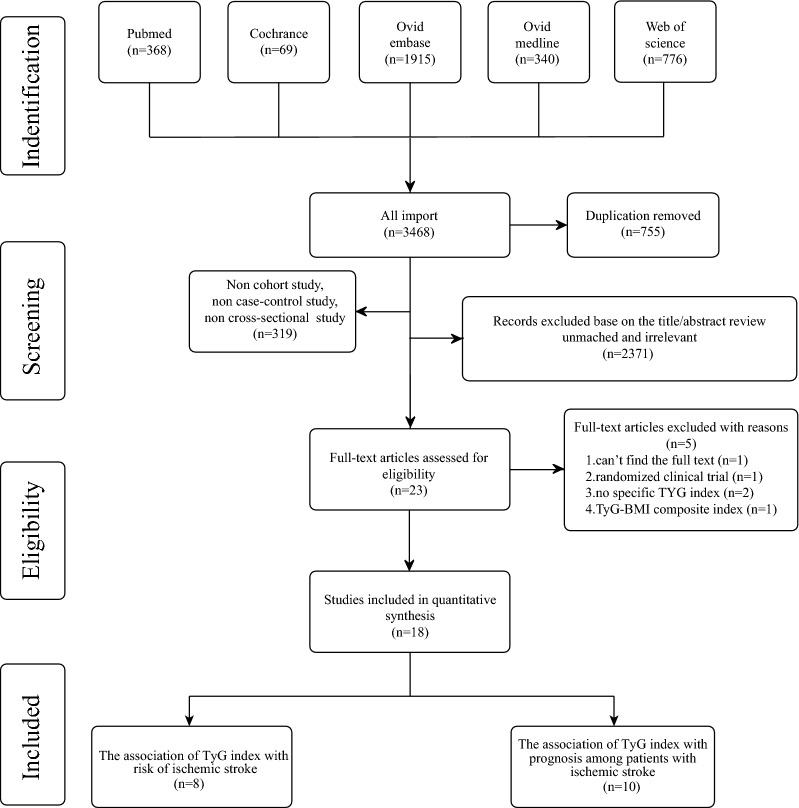
The flowchart of research screening

A flow sheet of the literature selection process can be visualized in Fig. 1. Through the database searches, according to the established search strategy, a total of 3468 articles were selected for subsequent filtering. Of these, 755 duplicate articles were excluded and 319 articles were removed because of non-cohort studies, non-case–control studies, and non-cross-sectional studies. After checking the title and abstract of each paper and excluding the inconsistent literature types, 23 studies were found to be pertinent to the research topic. Among them, one article was excluded due to inaccessibility of the full text [27], and four articles were further excluded after detailed review of the full text [28–31] and were found to not meet the appropriate inclusion criteria. A total of 18 original studies were finalized for inclusion in the meta-analysis, of which 8 studies [19, 21, 29, 31–35] were based on the risk of IS and 10 studies [22, 23, 36–43] were based on the prognosis among IS patients.

### Summary of studies

The characteristics of the included studies are summarized in Table 1. 59, 2635 individuals were recorded in the 18 studies, including 38,301 IS patients and 554,334 in the general population (defined as those age ≥ 18 years who have not had a stroke). There were three cross-sectional studies [29, 38, 40] and fifteen cohort studies [19, 21–23, 31–37, 39, 41–43], consisting of prospective and retrospective designs, and no randomized controlled trials. The population of the original studies were mainly from China [19, 21–23, 29, 31–34, 36, 39, 41, 43], with three from Korea [37, 38, 40], one from Singapore [42], and one from European [35]. The primary data sources included the eICU (emergency intensive care unit) Collaborative Research Database [36], the China National Stroke Registry [22, 39, 41], the UK Biobank cohort [35], the hospital stroke-related information [21, 23, 34, 37, 38, 40, 42, 43], and the community-based or rural population studies [19, 29, 31–33]. IS patients included in the analysis had a larger proportion of males, with a mean age greater than 60 years. The diagnosis of stroke consists of the clinical diagnosis, imaging diagnosis, and comprehensive diagnosis. The TyG index of most articles was calculated by the general formula, which is ln[triglyceride (mg/dL) ×fasting blood glucose (mg/dL)/2] [44]. Twelve studies used TyG index quartiles [19, 22, 29, 31–36, 39, 41, 43], two used TyG index tertiles [23, 40] three others followed a TyG index dichotomy [21, 37, 42] and the remaining study did not mention specific TyG index conditions [38]. The main adverse clinical outcomes observed in the ischemic stroke patient population included stroke recurrence, all-cause mortality, impairment in functional outcomes, deterioration in neurological function. The observed outcomes in the general population were the initial occurrence of stroke.

**Table 1:** Characteristics of studies inclcuded in the meta-analysis

Study	Country	Study design	Male%	Age (mean ± sd)	Study time	Participants	TyG index condition	Type of outcome
The association of TyG index with risk of ischemic stroke								
Wenrui S 2019	China	cross-sectional	40.20%	59.95 ± 10.07	8 M	10,900 from rural areas of northeast China	TyG I	IS Incidence
Anxin Wang 2021	China	prospective cohort	79.62%	51.38 ± 11.45	11.02 Y	97,653 from the Kailuan community in Tangshan City, China	TyG I	IS Incidence
Yang Zhao 2021	China	prospective cohort	40.88%	53.93 ± 11.19	6 Y	11,777 from the Rural Chinese Cohort Study	TyG I	IS Incidence
Qian Liu 2022	China	prospective cohort	79.61%	51.49 ± 12.53	10.33 Y	96,541 from the Kailuan community in Tangshan City, China	TyG I	IS Incidence
Qi Zhao 2021	China	retrospective cohort	73.70%	59.70 ± 9.30	4 Y	1510 individuals diagnosed with NSTE-ACS	TyG II	IS Incidence
Shucheng Si 2021	European	prospective cohort	43.01%	56.00 ± 8.02	4 Y	273,368 from the UK Biobank cohort	TyG I	IS Incidence
Longlong Hu 2022	China	prospective cohort	52.76%	68.77 ± 6.19	1.72 Ys	8487 from the China H-type Hypertension Registry Study	TyG I	IS Incidence
Xianxuan Wang 2022	China	prospective cohort	76.08%	49.03 ± 11.84	9 Y	54,098 from the Kailuan community in Tangshan City, China	TyG I	IS Incidence
The association of TyG index with prognosis among patients with ischemic stroke								
Yimo Zhou 2020	China	cohort	63.48%	64.83 ± 11.90	1 Y	16,310 from the China National Stroke Registry II	TyG I	Stroke recurrence, All-cause mortality, Poor functional outcome, Neurologic worsening
Bingjun Zhang 2020	China	retrospective observational	51.60%	66.30 ± 14.20	2 Y	4570 from the eICU Collaborative Research Database (208 United States hospitals)	TyG I	All-cause mortality
Minwoo Lee 2021	Korea	retrospective observational	59.00%	69.50 ± 12.40	4 Y	183 patients from three university-affiliated hospitals	TyG II	Poor functional outcome
Ki-Woong N (a) 2021	Korea	retrospective cross-sectional	59.30%	69.04 ± 11.24	4 Y	305 from the Seoul National University Hospital	TyG IV	Neurologic worsening
Zongyi Hou 2021	China	prospective cohort	63.30%	64.83 ± 12.20	1 Y	12,964 from the China National Stroke Registry II	TyG I	Stroke recurrence, Poor functional outcome, All-cause mortality
Ki-Woong N (b) 2021	Korea	retrospective cross-sectional	58.52%	72.03 ± 5.60	7 Y	176 from the Seoul National University Boramae Medical Center	TyG III	Stroke recurrence
Xiaomeng Yang 2022	China	cohort	62.97%	62.66 ± 14.60	1 Y	1226 from the Abnormal Glucose Regulation in Patients with Acute Stroke across China registry	TyG I	Stroke recurrence, All-cause mortality
Sheng-Feng Lin 2022	China	multicenter prospective cohort	63.68%	69.09 ± 12.23	12 Y	914 clinical data from 30 hospitals in Taiwan were collected and registered in the TTT-AIS registry	TyG III	Poor functional outcome, All-cause mortality
Emma M. S.Toh 2022	Singapore	retrospective cohort	61.50%	65.35 ± 15.60	11.75 Y	698 from a comprehensive stroke center	TyG II	All-cause mortality, Poor functional outcome
Fang Wang 2022	China	retrospective cohort	67.20%	70.70 ± 5.94	1 Y	955 from the Nanjing Stroke Registry Program	TyG I	Stroke recurrence

IS ischemic stroke, NSTE-ACS: non-ST-segment elevation acute coronary syndrome, *TTT-AIS* The Taiwan thrombolytic therapy for acute ischemic Stroke, *M* month, *Y* year, *Ys* years, *TyG I* TyG index quartiles*, TyG II* TyG index dichotomized, *TyG III* TyG index is divided into three categories, *TyG IV* TyG index (Uncategorized, count data)

### Overall assessment of evidence quality

The cohort studies' quality assessments resulted in NOS scores of 9 for eight studies, 8 for two studies, 7 for one study, and 6 for four studies. Their scores differed in the options of whether confounders were adequately adjusted and follow-up completeness. There were 11 high-quality studies, 4 moderate-quality studies, and no low-quality studies. In the cross-sectional study, one study had an AHRQ score of 10 and two other AHRQ scores of 8, which were unclear on the items of whether subjective and objective indicators were isolated, whether confounders were adequately adjusted, and the completeness of data and follow-up. All three included cross-sectional studies were considered of high quality. Specific scores of each study are listed in Additional files 2, 3: Table S2, S3.

### The association of TyG index with risk of IS in the general population

**Fig. 2:**
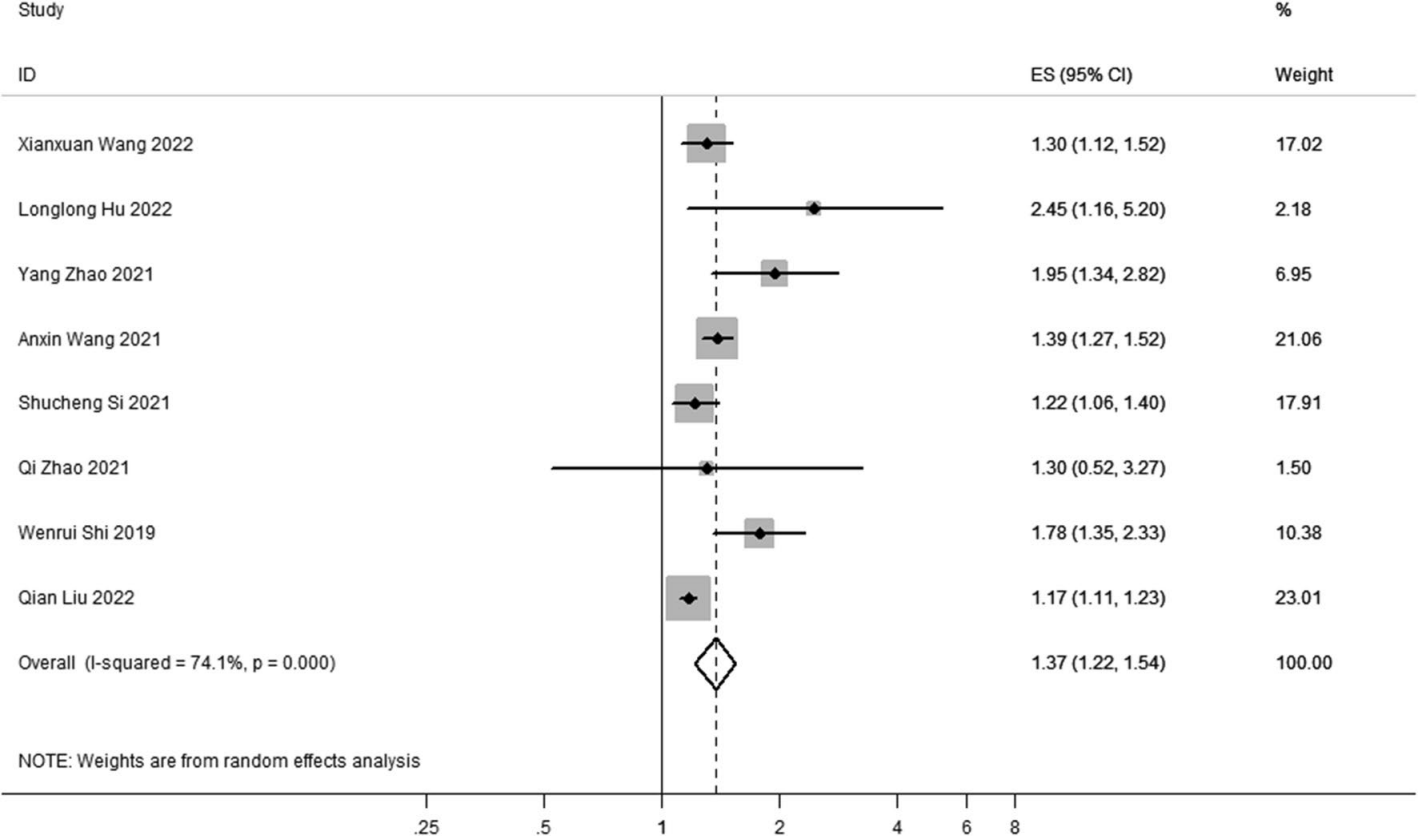
Forest plot of the association between TyG index and risk of ischemic stroke

There were 554,334 individuals in the selected eight studies of the general population. TyG index conditions used in the studies included TyG index quartiles and TyG index dichotomy. Compared to the general population with a lower TyG index, the general population with a higher TyG index had a 37.10% increased risk of IS (OR: 1.37; 95%CI 1.22–1.54). Since *I*^*2*^ = 74.1% and P = 0.000, a random effects model was used to aggregate the correlation effect values (Fig. 2).

The subgroup analyses were performed on covariates such as study design, country, the proportion of males, mean age, sample size, study time and participants. The detailed results are described in Table 2. Then, a univariate meta-regression analysis was used to examine the potential source of heterogeneity, and it was found that heterogeneity could be partly explained by sample size (studies with larger sample sizes reported a lower risk of IS development: β = − 0.382, SE = 0.122; p = 0.020). The remaining meta-regression analysis results are presented in Additional file 4: Table S4.

**Table 2:** Subgroup analysis of the meta-analysis for the risk of ischemic stroke

Analysis	Fixed-effect model		Random-effect model		I-squared (%)	P(Q-text)	Model
	Effect value (95% CI)	P (z-text)	Effect value (95% CI)	P (z-text)			
Country	1.245 1.196 1.296	< 0.001	1.371 1.220 1.540	< 0.001	74.10	< 0.001	Random model
China	1.248 1.196 1.301	< 0.001	1.421 1.234 1.637	< 0.001	77.80	< 0.001	Random model
European	1.220 1.062 1.402	0.005	1.220 1.062 1.402	0.005	–	–	–
Study desgin	1.245 1.196 1.296	< 0.001	1.371 1.220 1.540	< 0.001	74.10	< 0.001	Random model
Cohort study	1.236 1.186 1.287	< 0.001	1.320 1.182 1.475	< 0.001	70.60	0.002	Random model
Cross-sectional study	1.776 1.353 2.332	< 0.001	1.776 1.353 2.332	< 0.001	–	–	–
Male%	1.245 1.196 1.296	< 0.001	1.371 1.220 1.540	< 0.001	74.10	< 0.001	Random model
≥ 50%	1.230 1.178 1.283	< 0.001	1.300 1.144 1.477	< 0.001	72.50	0.006	Random model
< 50%	1.371 1.219 1.542	< 0.001	1.568 1.140 2.158	0.006	79.20	0.008	Random model
Mean age	1.245 1.196 1.296	< 0.001	1.371 1.220 1.540	< 0.001	74.10	< 0.001	Random model
≥ 65 years	2.450 1.157 5.187	0.019	2.450 1.157 5.187	0.019	–	–	-
< 65 years	1.243 1.194 1.294	< 0.001	1.349 1.205 1.511	< 0.001	74.90	0.001	Random model
Sample size	1.245 1.196 1.296	< 0.001	1.371 1.220 1.540	< 0.001	74.10	< 0.001	Random model
≥ 50,000	1.226 1.177 1.277	< 0.001	1.262 1.147 1.390	< 0.001	73.40	0.010	Random model
< 50,000	1.843 1.501 2.264	< 0.001	1.843 1.501 2.264	< 0.001	0.00	0.740	Fixed model
Study time	1.245 1.196 1.296	< 0.001	1.371 1.220 1.540	< 0.001	74.10	< 0.001	Random model
≥ 5 years	1.234 1.183 1.288	< 0.001	1.334 1.161 1.532	< 0.001	82.50	0.001	Random model
< 5 years	1.340 1.187 1.512	< 0.001	1.528 1.119 2.086	0.008	64.10	0.039	Random model
Participants	1.245 1.196 1.296	< 0.001	1.371 1.220 1.540	< 0.001	74.10	< 0.001	Random model
Community	1.243 1.194 1.294	< 0.001	1.352 1.203 1.519	< 0.001	79.10	< 0.001	Random model
Hospital	1.904 1.065 3.404	0.030	1.895 1.033 3.476	0.039	7.80	0.298	Fixed model
High quality	1.245 1.196 1.296	< 0.001	1.371 1.220 1.540	< 0.001	74.10	< 0.001	Random model
Yes	1.239 1.188 1.291	< 0.001	1.373 1.195 1.579	< 0.001	83.0	< 0.001	Random model
No	1.332 1.150 1.544	< 0.001	1.430 1.040 1.966	0.028	24.1	0.268	Fixed model

CI: confidence intervals

To test the publication bias of the included studies, we used Begg's test (z = 0.12, P = 0.902) and Egger’s test (t = 0.03, P = 0.040), with our results suggesting that there was possible publication bias in the included studies. At the same time, a visual funnel plot revealed that individual studies tilted slightly to the right side of the funnel. Further analysis was then performed by using the clip-and-complement method, indicating that there might be one grey publications (potential/unpublished/negative studies) which had not been found. After cutting and repairing, the distribution of individual studies on both sides of the funnel was found to be more balanced (Additional file 8: Figure S1). Sensitivity analyses were also performed to determine the stability and reliability of the results and the extent in which individual studies influenced the results. The effect size after excluding individual studies was in good agreement with the total combined effect size and confidence interval (OR 1.37, 95% CI 1.22–1.54), indicating that our results were robust to a certain extent (Additional file 9: Figure S2).

### The association of TyG index with prognosis among IS patients

**Fig. 3:**
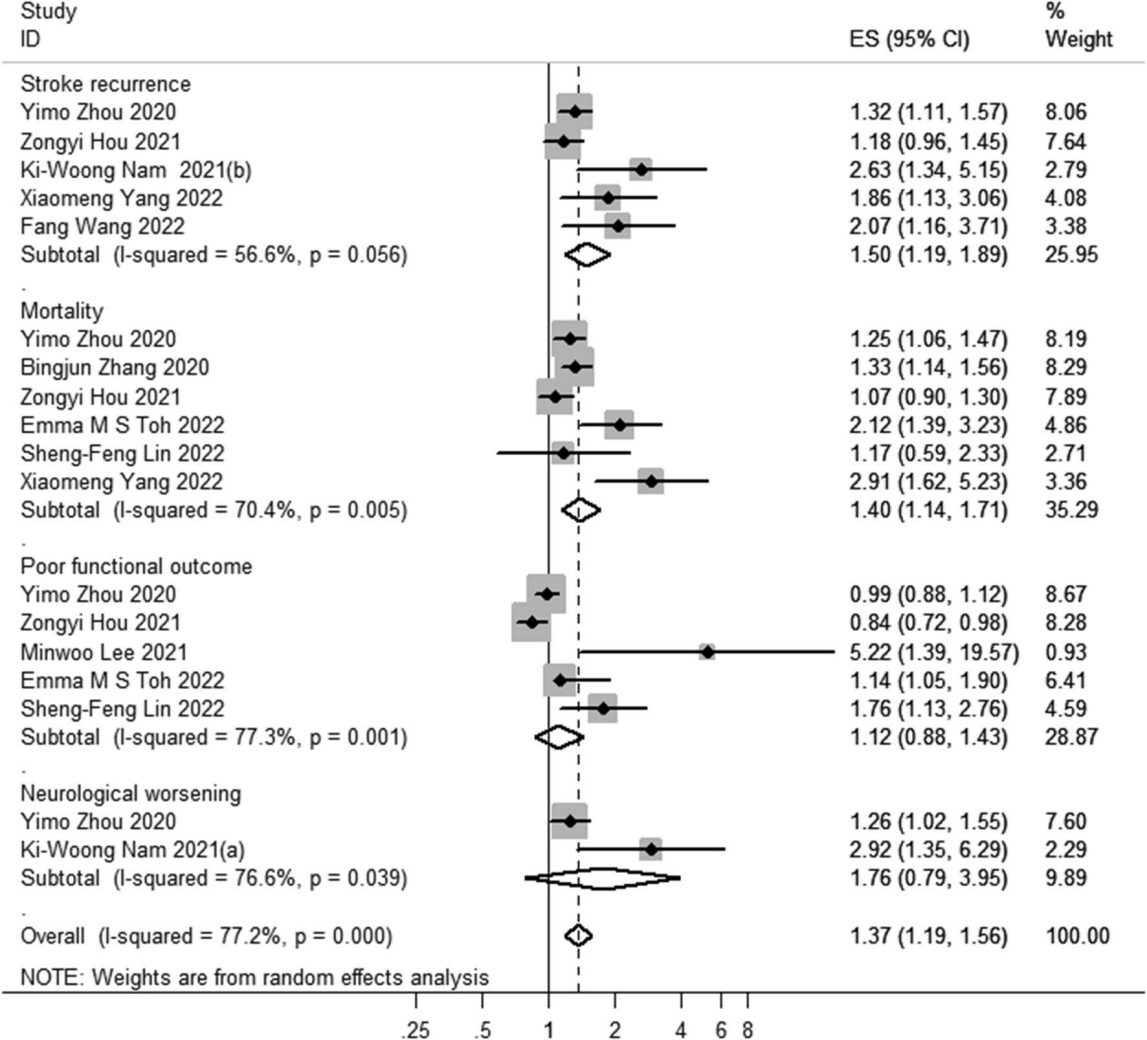
Forest plot of the TyG index association with prognosis among IS patients

A total sample of 38,301 cases was included in the ten IS population studies. In the IS patients, adverse clinical outcomes included stroke recurrence, all-cause mortality, impaired functional outcome, and neurological deterioration, and the same clinical outcomes were analyzed with a combined effect size. Compared with the IS population with a lower TyG index, IS patients with a higher TyG index had a higher risk of stroke recurrence (OR 1.50; 95% CI 1.19–1.89), increased risk of all-cause mortality (OR 1.40; 95% CI 1.14–1,71), while no association was found in the effect value combination of poor functional outcome (OR: 1.12; 95% CI 0.88–1.43) and neurological worsening (OR,1.76; 95% CI 0.79–3.95). Notably, Yimo Zhou's study [22] (OR 1.26; 95% CI 1.02–1.55) and Ki-Woong Nam's study [37] (OR: 2.92; 95% CI 1.35–6.29) reported that poor prognosis of neurological deterioration was associated with TyG index. Due to *I*^2^ = 77.20% and P = 0.000, a random effects model was used to aggregate the correlation effect sizes. Subsequently, the effect sizes of all clinical outcomes in ischemic stroke patients were combined, indicating that higher TyG index was associated with an increased risk of adverse clinical outcomes in ischemic stroke patients (OR 1.37; 95% CI 1.19–1.56) (Fig. 3).

In addition, we performed a subgroup analysis and meta-regression on prognosis outcomes available with more than three data items, such as stroke recurrence, all-cause mortality, and impaired functional outcome (See Additional files 5, 6: Table S5, S6 for detailed information). The results showed no publication bias in the included studies on all-cause mortality and impaired functional outcome; however there was a possible risk of publication bias in the studies on stroke recurrence (See Additional file 7: Table S7 for detailed information). We also performed sensitivity analyses on their outcomes to identify the stability of the pooled outcomes of these adverse clinical outcomes (See Additional files 10, 11, 12 Fig. S3, S4 S5 for detailed information).

## Discussion

This is the first systematic review and meta-analysis to integrate the association of the TyG index with IS risk in the general population and adverse clinical outcomes in IS patients. We found that there was a positive association between TyG index and increased risk of incident IS and this relationship remained in a stable state even as covariates changed. Meanwhile, TyG index was significantly associated with an increased risk of stroke recurrence and mortality, though not with poor functional outcome and neurologic worsening in stroke patients.

A previous meta-analysis reported an association between TyG index and stroke [18]. This study demonstrated that an increased TyG index was an independent risk factor for stroke; however, this phenomenon was not found in a subgroup analysis for those with hemorrhagic stroke. They also illustrated that TyG index was linearly associated with stroke events only limited to patients with type 2 diabetes or acute coronary syndrome [18]. In contrast, for our study, we only focused on IS in stroke specific studies and those focusing on the general population. Furthermore, we found that the TyG index may be an important predictor of specific clinical adverse outcomes in the setting of IS. The type of study, country of origin, study length, sample size, and definition of the TyG index were also found to have an impact on the association between IS and the TyG index. Thus, lending further data to the current literature as previous studies did not account for these variables.

The TyG index is known as a low-cost and efficient biomarker of insulin resistance (IR) [11], and IR plays an important role in the pathogenesis of IS through various potential mechanisms. IR can interfere with insulin signaling, enhance chronic systemic inflammation, reduce insulin sensitivity, and increase foam cell formation, thereby accelerating the formation of atherosclerosis and advanced plaques [45–49]. IR can also affect the metabolism of insulin-like growth factor-1 (IGF-1), insulin-like growth factor-2 (IGF-2) [50], cyclic guanosine monophosphate (cGMP), and nitric oxide(NO) [51], thus playing key roles in platelet adhesion, activation, and aggregation [52–54]. These pathways lead to vascular occlusion and in the pathogenesis of IS [54–56]. Furthermore, since IR may affect the cerebrovascular reserve (CVR) through the Baylis effect (myogenic mechanism), chemical, neuronal, and metabolic mechanisms [57–59], resulting in impaired cerebral perfusion hemodynamics, this may lead to the hemodynamic disturbances of cerebral perfusion during acute IS [60, 61]. Given these factors, the TyG index reflects the IR level, which can indirectly be used to predict IS.

In both past and present studies, it is well established that elevated IR is a correlative predictor of IS [28–32, 62, 63]. In the general population, the relationship between TyG index and the risk of IS is clear as suggested by our results. However, whether IR can truly guide the clinical outcome of IS patients is controversial. Our analysis showed that TyG index was valuable for predicting recurrent stroke and mortality, but there was no significant correlation found between functional status decline and neurological impairment. Interestingly, previous large prospective studies and meta-analyses supported a significant increase in the risk of stroke recurrence, and all-cause mortality was observed during the 12-month follow-up period, which significantly correlated with IR [22, 64, 65]. Similarly, the interpretation of whether the TyG index can predict poor function or neurological deficits need to be carefully interpreted and studied as well. For example, both Yimo Zhou [22] and Ki-Woong Nam [38] reported that TyG index can predict neurologic worsening, however the combined data was incongruent with this conclusion. This is likely due to unaccounted for heterogeneity. Notably, the severity of functional and neurological deficits of IS affected by many factors, such as robust secondary prevention methods, the presence of CYP2C19 polymorphisms, adequate rehabilitation strategies, excellent family support, the severity of IS itself, and type of emergency treatment approaches [66–75]. In our meta-analysis, we analyzed the source of heterogeneity via meta-regression analyses, and discovered that variables including country, study design, TyG index conditions, sample size, and follow-up time may each be responsible for the source of heterogeneity. In the future, more accurate TyG measurement methods, cross-validated research designs, larger sample sizes, and multicenter studies are required to further analyze and resolve this conclusion.

Our study had several advantages: First, we divided the study into two types of samples: the general population and the stroke population, which is of great significance for the guidance and application of the TyG index in clinical practice. Second, the association between TyG index and stroke or stroke outcomes was not affected by any single inclusion factor or participant selection according to our sensitivity analysis and subgroup analysis. All of these facets lent evidence to the stability and credibility of our conclusions. Although we conducted study selection, data extraction and quality assessment in strict accordance with established standards, the present study had several limitations. To begin with, most of the literature data are from Asian countries, such as China, South Korea and Singapore, and only one study is from Europe. Although the European research conclusion is consistent with ours, it is questionable whether these results would remain congruent across other countries, regions, and ethnic groups. Additionally, the calculation of TyG index depends on triglyceride and glucose levels which are measured in a non-standardized approach at different institutions. Therefore, measurement errors caused by different detection conditions and levels cannot be fully excluded from the analysis, which may weaken the reliability of the results in our meta-analysis. Last but not least, in the calculation of publication bias, there is a possible inconsistency between the Begg's test and the Egger's test. Although we used different methods to recalculate, it still suggests that the results of the two are different, which means that there is still potential publication bias.

## Conclusion and future directions

In conclusion, our meta-analysis reveals that TyG index has potential value in optimizing risk stratification for IS in the general population. Furthermore, there is a significant association between TyG index and major adverse outcomes of strokes. A higher TyG index confers a higher risk of stroke recurrence and risk of death. Notably, no association was found in the effect size combination of poor functional outcomes or neurological worsening. Finally, our subgroup analysis observed that variables such as country, type of stroke, participant, and sample size all contributed an impact to the heterogeneity of the results. Our results suggest that attention should be paid to TyG index screening in the management of stroke patients in the future. Early detection of stroke risk and potential clinical adverse outcomes can delay disease progression and reduce social and economic burdens. TyG index detection is convenient and affordable, however there are relatively few studies on TyG index specifically in relation to IR index and stroke currently. As such, more prospective cohort studies with large sample sizes and multicenter trials using TyG index as a predictor should be carried out to advance our clinical management and utilization of this biomarker in stroke patients. Furthermore, well-designed basic science studies are needed to explore the underlying mechanisms and relationship of TyG index and stroke pathophysiology and sequelae.

## Supplementary Information

**Additional file 1: Table S1.** Search Strategy

**Additional file2: Table S2.** The Newcastle-Ottawa Quality Assessment Scale score for cohort studies.

**Additional file 3: Table S3.** The Agency for Healthcare Research and Quality cross-sectional study evaluation criteria.

**Additional file 4: Table S4.** Table S4. Univariate meta-regression analysis for the TyG index association with ischemic stroke risk.

**Additional file 5: Table S5.** Subgroup analysis for the association of TyG index with prognosis among patients with ischemic stroke.

**Additional file 6: Table S6.** Univariate meta-regression analysis for the TyG index association with prognosis among patients with ischemic stroke.

**Additional file 7 Table S7.** The publication bias of TyG index with prognosis among patients with ischemic stroke.

**Additional file 8: Fig S1.** The publication bias assessment of the TyG index association with ischemic stroke risk.

**Additional file 9: Fig S2.** The sensitivity analysis for the TyG index association with ischemic stroke risk.

**Additional file 10: Fig S3.** Sensitivity analysis for the association of TyG index with Mortality among patients with ischemic stroke.

**Additional file 11: Fig S4.** Sensitivity analysis for the association of TyG index with Stroke recurrence among patients with ischemic stroke.

**Additional file 12: Fig S5.** Sensitivity analysis for the association of TyG index with Poor functional outcome among patients with ischemic stroke.

## Data Availability

Not applicable.

## Abbreviations

AF: Atrial fibrillation
AHRQ: The agency for healthcare research and quality
CI: Confidence intervals
cGMP: Cyclic guanosine monophosphate
CT: Computed tomography
CVD: Cardiovascular disease
CVR: The cerebrovascular reserve
ES: Effect size
HS: Hemorrhagic strokes
HIEC: Hyperinsulinemic-euglycemic clamp
IGF-1: Insulin-like growth factor-1
IGF-2: Insulin-like growth factor-2
IR: Insulin resistance
IS: Ischemic stroke
MetS: Metabolic syndrome
MRI: Magnetic resonance imaging
NO: Nitric oxide
NOS: Newcastle–Ottawa quality assessment scale
PRISMA: Preferred reporting items for systematic reviews and meta-analyses
PVC: Premature ventricular complexes
TyG: Triglyceride and glucose
