# Supplementary material for: The impact of triglyceride-glucose index on ischemic stroke: a systematic review and meta-analysis
Source: Cardiovasc Diabetol. 2023 Jan 6;22:2. doi: 10.1186/s12933-022-01732-0 (PMC9825038; doi:10.1186/s12933-022-01732-0)
Supplement: Supplementary file 1 — Additional file 1: Table S1. Search Strategy [file 12933_2022_1732_MOESM1_ESM.docx]

**Additional file 1: Table S1. Search Strategy**

| **Pubmed** | | | **Cochrane library** | | | **Ovid MEDLINE** | | | **Ovid EMBASE** | | | **Web of Science** | | |
| --- | --- | --- | --- | --- | --- | --- | --- | --- | --- | --- | --- | --- | --- | --- |
| Search | Query | Items | Search | Query | Items | Search | Query | Items | Search | Query | Items | Search | Query | Items |
| #1 | ((triglyceride glucose index[Title/Abstract]) OR (TyG index[Title/Abstract])) OR (TyG[Title/Abstract]) | 580 | #1 | (triglyceride glucose index OR TyG index OR TyG):ti,ab,kw | 1434 | #1 | (triglyceride glucose index or TyG index or TyG).ab,kw,ti. | 575 | #1 | (triglyceride glucose index or TyG index or TyG).ab,kw,ti. | 758 | #1 | TOPIC:(triglyceride glucose index OR TyG index OR TyG) | 26,717 |
| #2 | Triglycerides[Mesh] | 81,098 | #2 | MeSH descriptor: [Triglycerides] Explode all trees | 6592 | #2 | exp Triglycerides/ | 81,101 | #2 | exp Triglycerides/ | 223,699 | #2 | TOPIC:(Triglycerides[Mesh]) OR TOPIC:(Triacylglycerol OR Triglyceride* OR TG OR TAG) | 1,164,375 |
| #3 | (((Triacylglycerol[Title/Abstract]) OR (Triglyceride*[Title/Abstract])) OR (TG[Title/Abstract])) OR (TAG[Title/Abstract]) | 21,3835 | #3 | (Triacylglycerol OR Triglyceride* OR TG OR TAG):ti,ab,kw | 31,317 | #3 | (Triacylglycerol or Triglyceride* or TG or TAG).ab,kw,ti. | 210,388 | #3 | (Triacylglycerol or Triglyceride* or TG or TAG).ab,kw,ti. | 293,370 | #3 | TOPIC:(Glucose[Mesh]) OR TOPIC:(GLU OR Glc OR Amylaceum OR dextrose OR D-Glucose OR Dextrose, Anhydrous OR Glucose,(L)-Isomer OR L-Glucose OR Glucose Monohydrate) | 238,851 |
| #4 | #2 OR #3 | 24,1443 | #4 | #2 OR #3 | 31,329 | #4 | #2 OR #3 | 238,059 | #4 | #2 OR #3 | 373,237 | #4 | #2 AND #3 | 7,911 |
| #5 | Glucose[Mesh] | 322,968 | #5 | MeSH descriptor: [Glucose] Explode all trees | 19,621 | #5 | exp Glucose/ | 322,984 | #5 | exp Glucose/ | 448,574 | #5 | #1 OR #4 | 33,504 |
| #6 | ((((((((GLU[Title/Abstract]) OR (Glc[Title/Abstract])) OR (Amylaceum[Title/Abstract])) OR (dextrose[Title/Abstract])) OR (D-Glucose[Title/Abstract])) OR (Dextrose, Anhydrous[Title/Abstract])) OR (Glucose, (L)-Isomer[Title/Abstract])) OR (L-Glucose[Title/Abstract])) OR (Glucose Monohydrate[Title/Abstract]) | 77,433 | #6 | (GLU OR Glc OR Amylaceum OR dextrose OR D-Glucose OR Dextrose, Anhydrous OR Glucose,L-Isomer OR L-Glucose OR Glucose Monohydrate):ti,ab,kw | 3854 | #6 | (GLU or Glc or Amylaceum or dextrose or D-Glucose or Dextrose, Anhydrous or Glucose,L-Isomer or L-Glucose or Glucose Monohydrate).ab,kw,ti. | 76,395 | #6 | (GLU or Glc or Amylaceum or dextrose or D-Glucose or Dextrose, Anhydrous or Glucose,L-Isomer or L-Glucose or Glucose Monohydrate).ab,kw,ti. | 90,901 | #6 | TOPIC:(Stroke[Mesh]) OR TOPIC:(Apoplexy OR Stroke* OR Apoplexia OR Cerebrovascular Accident* OR CVA* OR Cerebrovascular Apoplexy OR Apoplexy, Cerebrovascular OR Vascular Accident*, Brain OR Brain Vascular Accident* OR Cerebrovascular Stroke* OR Stroke*, Cerebrovascular OR Cerebral Stroke* OR Stroke*, Cerebral OR Stroke*, Acute OR Acute Stroke* OR Cerebrovascular Accident*, Acute OR Acute Cerebrovascular Accident*) | 806,138 |
| #7 | #5 OR #6 | 385,328 | #7 | #5 OR #6 | 22,766 | #7 | #5 OR #6 | 384,630 | #7 | #5 OR #6 | 515,253 | #7 | #5 AND #6 | 776 |
| #8 | #4 AND #7 | 27,618 | #8 | #4 AND #7 | 3467 | #8 | #4 AND #7 | 27,524 | #8 | #4 AND #7 | 67,188 |  |  |  |
| #9 | #1 OR #8 | 27,890 | #9 | #1 OR #8 | 4506 | #9 | #1 OR #8 | 27,793 | #9 | #1 OR #8 | 67,513 |  |  |  |
| #10 | Stroke* [Mesh] | 208,343 | #10 | MeSH descriptor: [Stroke] explode all trees | 11,217 | #10 | exp Stroke/ | 156,917 | #10 | exp Stroke/ | 261,106 |  |  |  |
| #11 | ((((((((((((((((Apoplexy[Title/Abstract]) OR (Stroke*[Title/Abstract])) OR (Apoplexia[Title/Abstract])) OR (Cerebrovascular Accident*[Title/Abstract])) OR (CVA* (Cerebrovascular Accident)[Title/Abstract])) OR (Cerebrovascular Apoplexy[Title/Abstract])) OR (Apoplexy, Cerebrovascular[Title/Abstract])) OR (Vascular Accident*, Brain[Title/Abstract])) OR (Brain Vascular Accident*[Title/Abstract])) OR (Cerebrovascular Stroke*[Title/Abstract])) OR (Stroke*, Cerebrovascular[Title/Abstract])) OR (Cerebral Stroke*[Title/Abstract])) OR (Stroke*, Cerebral[Title/Abstract]) ) OR (Stroke*, Acute[Title/Abstract])) OR (Acute Stroke*[Title/Abstract])) OR (Cerebrovascular Accident*, Acute[Title/Abstract])) OR (Acute Cerebrovascular Accident*[Title/Abstract]) | 317,440 | #11 | (Apoplexy OR Stroke* OR Apoplexia OR Cerebrovascular Accident* OR CVA* (Cerebrovascular Accident) OR Cerebrovascular Apoplexy OR Apoplexy, Cerebrovascular OR Vascular Accident*, Brain OR Brain Vascular Accident* OR Cerebrovascular Stroke* OR Stroke*, Cerebrovascular OR Cerebral Stroke* OR Stroke*, Cerebral OR Stroke*, Acute OR Acute Stroke* OR Cerebrovascular Accident*, Acute OR Acute Cerebrovascular Accident*):ti,ab,kw | 64,796 | #11 | (Apoplexy or Stroke* or Apoplexia or Cerebrovascular Accident* or CVA* or Cerebrovascular Apoplexy or Apoplexy, Cerebrovascular or Vascular Accident*, Brain or Brain Vascular Accident* or Cerebrovascular Stroke* or Stroke*, Cerebrovascular or Cerebral Stroke* or Stroke*, Cerebral or Stroke*, Acute or Acute Stroke* or Cerebrovascular Accident*, Acute or Acute Cerebrovascular Accident*).ab,kw,ti. | 300,941 | #11 | (Apoplexy or Stroke* or Apoplexia or Cerebrovascular Accident* or CVA* or Cerebrovascular Apoplexy or Apoplexy, Cerebrovascular or Vascular Accident*, Brain or Brain Vascular Accident* or Cerebrovascular Stroke* or Stroke*, Cerebrovascular or Cerebral Stroke* or Stroke*, Cerebral or Stroke*, Acute or Acute Stroke* or Cerebrovascular Accident*, Acute or Acute Cerebrovascular Accident*).ab,kw,ti. | 482,279 |  |  |  |
| #12 | #10 OR #11 | 383,145 | #12 | #10 OR #11 | 65,222 | #12 | #10 OR #11 | 337,561 | #12 | #10 OR #11 | 543,048 |  |  |  |
| #13 | #9 AND #12 | 368 | #13 | #9 AND #12 | 69 | #13 | #9 AND #12 | 340 | #13 | #9 AND #12 | 1915 |  |  |  |
