# Supplementary material for: The impact of triglyceride-glucose index on ischemic stroke: a systematic review and meta-analysis
Source: Cardiovasc Diabetol. 2023 Jan 6;22:2. doi: 10.1186/s12933-022-01732-0 (PMC9825038; doi:10.1186/s12933-022-01732-0)
Supplement: Supplementary file 2 — Additional file2: Table S2. The Newcastle-Ottawa Quality Assessment Scale score for cohort studies. [file 12933_2022_1732_MOESM2_ESM.docx]

**Supplementary table 2. The Newcastle-Ottawa Quality Assessment Scale score for cohort studies.**

| **Study** | **Selection (1)** | | | | **Comparability (2)** | **Outcome (3)** | | | **Score** |
| --- | --- | --- | --- | --- | --- | --- | --- | --- | --- |
|  |  |  |  |  |  |  |  |  |  |
|  | Representativeness of the exposed cohort | Selection of the non-exposed cohort | Ascertainment of exposure | Demonstration that outcome of Interest was not Present at start of study | Comparability of cohorts on the basis of the design or analysis | Assessment of outcome | Was follow-up long enough for outcomes to occur | Adequacy of follow up of cohorts |  |
| Yimo Zhou 2020 | 1 | 1 | 1 | 1 | 2 | 1 | 1 | 1 | 9 |
| Bingjun Zhang 2020 | 1 | 1 | 1 | 1 | 2 | 1 | 1 | 1 | 9 |
| Minwoo Lee 2021 | 0 | 1 | 1 | 1 | 1 | 1 | 0 | 1 | 6 |
| Anxin Wang 2021 | 1 | 1 | 1 | 1 | 2 | 1 | 1 | 1 | 9 |
| Yang Zhao 2021 | 1 | 1 | 1 | 1 | 2 | 1 | 1 | 1 | 9 |
| Zongyi Hou 2021 | 1 | 1 | 1 | 1 | 1 | 1 | 1 | 1 | 8 |
| Xiaomeng Yang 2022 | 1 | 1 | 1 | 0 | 1 | 1 | 0 | 1 | 6 |
| Sheng-Feng Lin 2022 | 1 | 1 | 1 | 1 | 2 | 1 | 1 | 1 | 9 |
| Emma M. S.Toh 2022 | 1 | 1 | 1 | 1 | 1 | 1 | 1 | 0 | 7 |
| Qian Liu 2022 | 1 | 1 | 1 | 1 | 2 | 1 | 1 | 1 | 9 |
| Qi Zhao 2021 | 0 | 1 | 1 | 1 | 1 | 1 | 1 | 0 | 6 |
| Shucheng Si 2021 | 1 | 1 | 1 | 1 | 1 | 1 | 1 | 1 | 8 |
| Longlong Hu 2022 | 0 | 1 | 1 | 0 | 2 | 1 | 1 | 0 | 6 |
| Xianxuan Wang 2022 | 1 | 1 | 1 | 1 | 2 | 1 | 1 | 1 | 9 |
| Fang Wang 2022 | 1 | 1 | 1 | 1 | 2 | 1 | 1 | 1 | 9 |
