# Supplementary material for: The impact of triglyceride-glucose index on ischemic stroke: a systematic review and meta-analysis
Source: Cardiovasc Diabetol. 2023 Jan 6;22:2. doi: 10.1186/s12933-022-01732-0 (PMC9825038; doi:10.1186/s12933-022-01732-0)
Supplement: Supplementary file 3 — Additional file 3: Table S3. The Agency for Healthcare Research and Quality cross-sectional study evaluation criteria. [file 12933_2022_1732_MOESM3_ESM.docx]

**Additional file 3: Table S3. The Agency for Healthcare Research and Quality cross-sectional study evaluation criteria.**

| **Study** | **（1）Define the source of information (survey , record review)** | **（2）List inclusion and exclusion criteria for exposed and unexposed subjects (cases and controls) or refer to previous publications** | **（3）Indicate time period used for identifying patients** | **（4）Indicate whether or not subjects were consecutive if not population-based** | **（5）Indicate if evaluators of subjective components of study were masked to other aspects of the status participants** | **（6）Describe any assessments undertaken for quality assurance purposes(e.g., test/retest of primary outcome measurements)** | **（7）Explain any patient exclusions from analysis** | **（8）Describe how confounding was assessed and/or controlled** | **（9）If applicable, explain how missing data were handled in the analysis** | **（10）Summarize patient response rates and completeness of data collection** | **（11） Clarify what follow-up, if any, was expected**  **and the percentage of patients for which incomplete data or follow-up was obtained** | **Score** |
| --- | --- | --- | --- | --- | --- | --- | --- | --- | --- | --- | --- | --- |
| Wenrui Shi 2019 | yes | yes | yes | yes | unclear | yes | yes | yes | yes | unclear | unclear | 8 |
| Ki-Woong Nam (a) 2021 | yes | yes | yes | yes | unclear | yes | yes | unclear | yes | unclear | yes | 8 |
| Ki‑Woong Nam (b) 2021 | yes | yes | yes | yes | unclear | yes | yes | yes | yes | yes | yes | 10 |
