# Supplementary material for: The impact of triglyceride-glucose index on ischemic stroke: a systematic review and meta-analysis
Source: Cardiovasc Diabetol. 2023 Jan 6;22:2. doi: 10.1186/s12933-022-01732-0 (PMC9825038; doi:10.1186/s12933-022-01732-0)
Supplement: Supplementary file 4 — Additional file 4: Table S4. Table S4. Univariate meta-regression analysis for the TyG index association with ischemic stroke risk. [file 12933_2022_1732_MOESM4_ESM.docx]

**Additional file 4: Table S4.** **Univariate meta-regression analysis for the TyG index association with ischemic stroke risk.**

| **Variable** | **β (95% CI)** | **SE** | **P** |
| --- | --- | --- | --- |
| Country: China vs European | 0.168 (-0.318, 0.653) | 0.198 | 0.430 |
| Study design: cohort study vs cross-sectional study | -0.301 (-0.800, 0.198) | 0.204 | 0.191 |
| Male%: ≥50% vs <50% | -0.141 (-0.505, 0.222) | 0.149 | 0.379 |
| Mean age: ≥65 years vs <65 years | 0.590 (-0.446, 1.627) | 0.423 | 0.213 |
| Mean age (continuous) | 0.026 (-0.010, 0.063) | 0.015 | 0.128 |
| Sample size: ≥50000 vs <50000 | -0.382 (-0.680, -0.085) | 0.122 | 0.020 |
| Study time: ≥5 years vs <5 years | -0.093 (-0.488, 0.303) | 0.162 | 0.588 |
| Participants: community vs other | -0.331 (-1.185, 0.523) | 0.349 | 0.380 |
| TyG index condition: TyG index quartile vs other | 0.064 (-1.296, 1.423) | 0.555 | 0.912 |
| High quality: Yes vs No | -0.023 (-0.490, 0.443) | 0.191 | 0.907 |

Abbreviations: TyG, Triglyceride-Glucose.
