# Supplementary material for: The impact of triglyceride-glucose index on ischemic stroke: a systematic review and meta-analysis
Source: Cardiovasc Diabetol. 2023 Jan 6;22:2. doi: 10.1186/s12933-022-01732-0 (PMC9825038; doi:10.1186/s12933-022-01732-0)
Supplement: Supplementary file 5 — Additional file 5: Table S5. Subgroup analysis for the association of TyG index with prognosis among patients with ischemic stroke. [file 12933_2022_1732_MOESM5_ESM.docx]

**Supplementary table 5. Subgroup analysis for the association of TyG index with prognosis among patients with ischemic stroke.**

|  | **Fixed-effect model** | | **Random-effect model** | |  |  |  |
| --- | --- | --- | --- | --- | --- | --- | --- |
| **Analysis** | **Effect value (95% CI)** | **P**  **(z-text)** | **Effect value (95% CI)** | **P**  **(z-text)** | **I-squared(%)** | **P(Q-text)** | **Model** |
| **Mortality** | | | | | | | |
| **Country** | 1.287 1.174 1.411 | <0.001 | 1.401 1.145 1.714 | 0.001 | 70.4% | 0.005 | **Random model** |
| China | 1.255 1.143 1.379 | <0.001 | 1.301 1.079 1.568 | 0.006 | 64.5% | 0.024 | **Random model** |
| Singapore | 2.120 1.391 3.232 | <0.001 | 2.120 1.391 3.232 | <0.001 | - | - | **-** |
| **Mean age** | 1.287 1.174 1.411 | <0.001 | 1.401 1.145 1.714 | 0.001 | 70.4% | 0.005 | **Random model** |
| ≥65 years | 1.395 1.210 1.608 | <0.001 | 1.499 1.080 2.080 | 0.015 | 54.6% | 0.111 | **Random model** |
| <65 years | 1.215 1.077 1.371 | 0.002 | 1.374 0.992 1.902 | 0.056 | 80.8% | 0.006 | **Random model** |
| **Sample size** | 1.287 1.174 1.411 | <0.001 | 1.401 1.145 1.714 | 0.001 | 70.4% | 0.005 | **Random model** |
| ≥5000 | 1.169 1.034 1.322 | 0.013 | 1.165 1.002 1.355 | 0.047 | 32.7% | 0.223 | **Fixed model** |
| <5000 | 1.453 1.265 1.669 | <0.001 | 1.724 1.172 2.536 | 0.006 | 70.3% | 0.018 | **Random model** |
| **Study time** | 1.287 1.174 1.411 | <0.001 | 1.401 1.145 1.714 | 0.001 | 70.4% | 0.005 | **Random model** |
| ≥5 years | 1.802 1.258 2.581 | 0.001 | 1.680 0.951 2.966 | 0.074 | 52.2% | 0.148 | **Random model** |
| <5 years | 1.257 1.143 1.383 | <0.001 | 1.318 1.074 1.617 | 0.008 | 73.2% | 0.011 | **Random model** |
| **High quality** | 1.287 1.174 1.411 | <0.001 | 1.401 1.145 1.714 | 0.001 | 70.4% | 0.005 | **Random model** |
| Yes | 1.261 1.149 1.384 | <0.001 | 1.290 1.097 1.517 | 0.002 | 56.9% | 0.055 | **Random model** |
| No | 2.910 1.620 5.229 | <0.001 | 2.910 1.620 5.229 | <0.001 | - | - | **-** |
| **Stroke recurrence** | | | | | | | |
| **Country** | 1.351 1.194 1.528 | <0.001 | 1.501 1.190 1.893 | 0.001 | 56.6% | 0.056 | **Random model** |
| China | 1.320 1.165 1.496 | <0.001 | 1.379 1.133 1.677 | 0.001 | 43.7% | 0.149 | **Fixed model** |
| Korea | 2.630 1.342 5.156 | 0.005 | 2.630 1.342 5.156 | 0.005 | - | - | **-** |
| **Mean age** | 1.351 1.194 1.528 | <0.001 | 1.501 1.190 1.893 | 0.001 | 56.6% | 0.056 | **Random model** |
| ≥65 years | 2.295 1.478 3.565 | <0.001 | 2.295 1.478 3.565 | <0.001 | 0.0% | 0.600 | **Fixed model** |
| <65 years | 1.291 1.136 1.468 | <0.001 | 1.305 1.103 1.544 | 0.002 | 31.3% | 0.233 | **Fixed model** |
| **Sample size** | 1.351 1.194 1.528 | <0.001 | 1.501 1.190 1.893 | 0.001 | 56.6% | 0.056 | **Random model** |
| ≥5000 | 1.258 1.102 1.437 | 0.001 | 1.258 1.102 1.437 | 0.001 | 0.0% | 0.401 | **Fixed model** |
| <5000 | 2.093 1.505 2.911 | <0.001 | 2.093 1.505 2.911 | <0.001 | 0.0% | 0.719 | **Fixed model** |
| **Study time** | 1.351 1.194 1.528 | <0.001 | 1.501 1.190 1.893 | 0.001 | 56.6% | 0.056 | **Random model** |
| ≥5 years | 2.630 1.342 5.156 | 0.005 | 2.630 1.342 5.156 | 0.005 | - | - | **-** |
| <5 years | 1.320 1.165 1.496 | <0.001 | 1.379 1.133 1.677 | 0.001 | 43.7% | 0.149 | **Fixed model** |
| **High quality** | 1.351 1.194 1.528 | <0.001 | 1.501 1.190 1.893 | 0.001 | 56.6% | 0.056 | **Random model** |
| Yes | 1.323 1.165 1.502 | <0.001 | 1.448 1.127 1.862 | 0.004 | 60.2% | 0.057 | **Random model** |
| No | 1.860 1.130 3.061 | 0.015 | 1.860 1.130 3.061 | 0.015 | - | - | **-** |
| **Poor functional outcome** | | | | | | | |
| **Country** | 0.979 0.896 1.070 | 0.638 | 1.121 0.878 1.431 | 0.359 | 77.3% | 0.001 | **Random model** |
| China | 0.956 0.871 1.050 | 0.347 | 1.034 0.802 1.334 | 0.794 | 80.5% | 0.006 | **Random model** |
| Korea | 5.220 1.391 19.587 | 0.014 | 5.220 1.391 19.587 | 0.014 | - | - | **-** |
| Singapore | 1.140 0.847 1.534 | 0.386 | 1.140 0.847 1.534 | 0.386 | - | - | **-** |
| **Mean age** | 0.979 0.896 1.070 | 0.638 | 1.121 0.878 1.431 | 0.359 | 77.3% | 0.001 | **Random model** |
| ≥65 years | 1.364 1.070 1.739 | 0.012 | 1.665 0.950 2.919 | 0.075 | 69.8% | 0.037 | **Random model** |
| <65 years | 0.930 0.845 1.023 | 0.136 | 0.918 0.780 1.080 | 0.301 | 63.7% | 0.097 | **Random model** |
| **Sample size** | 0.979 0.896 1.070 | 0.638 | 1.121 0.878 1.431 | 0.359 | 77.3% | 0.001 | **Random model** |
| ≥5000 | 0.930 0.845 1.023 | 0.136 | 0.918 0.780 1.080 | 0.301 | 63.7% | 0.097 | **Random model** |
| <5000 | 1.364 1.070 1.739 | 0.012 | 1.665 0.950 2.919 | 0.075 | 69.8% | 0.037 | **Random model** |
| **Study time** | 0.979 0.896 1.070 | 0.638 | 1.121 0.878 1.431 | 0.359 | 77.3% | 0.001 | **Random model** |
| ≥5 years | 1.302 1.017 1.667 | 0.036 | 1.370 0.900 2.086 | 0.142 | 60.3% | 0.112 | **Random model** |
| <5 years | 0.938 0.853 1.032 | 0.190 | 0.979 0.744 1.289 | 0.881 | 78.4% | 0.010 | **Random model** |
| **High quality** | 0.979 0.896 1.070 | 0.638 | 1.121 0.878 1.431 | 0.359 | 77.3% | 0.001 | **Random model** |
| Yes | 0.972 0.889 1.062 | 0.524 | 1.049 0.852 1.291 | 0.655 | 73.9% | 0.009 | **Random model** |
| No | 5.220 1.391 19.587 | 0.014 | 5.220 1.391 19.587 | 0.014 | - | - | **-** |

Abbreviations: TyG, Triglyceride-Glucose.
