# Supplementary material for: The impact of triglyceride-glucose index on ischemic stroke: a systematic review and meta-analysis
Source: Cardiovasc Diabetol. 2023 Jan 6;22:2. doi: 10.1186/s12933-022-01732-0 (PMC9825038; doi:10.1186/s12933-022-01732-0)
Supplement: Supplementary file 6 — Additional file 6: Table S6. Univariate meta-regression analysis for the TyG index association with prognosis among patients with ischemic stroke. [file 12933_2022_1732_MOESM6_ESM.docx]

**Additional file 6: Table S6.** **Univariate meta-regression analysis for the TyG index association with prognosis among patients with ischemic stroke.**

| **Variable** | **β (95% CI)** | **SE** | **P** |
| --- | --- | --- | --- |
| **Mortality** |  |  |  |
| Country: China vs Singapore | -0.476 (-1.530, 0.578) | 0.380 | 0.278 |
| Mean age: ≥65 years vs <65 years | 0.053 (-0.872, 0.978) | 0.333 | 0.882 |
| Mean age (continuous) | -0.104 (-0.356, 0.148) | 0.091 | 0.316 |
| Sample size: ≥5000 vs <5000 | -0.376 (-1.070, 0.319) | 0.250 | 0.208 |
| Study time: ≥5 years vs <5 years | 0.203 (-0.812, 1.217) | 0.365 | 0.609 |
| TyG index condition: TyG index quartiles vs TyG index is divided into three categories | -0.203 (-1.217, 0.812) | 0.365 | 0.609 |
| High quality: Yes vs No | -0.809 (-1.870, 0.252) | 0.382 | 0.102 |
| **Stroke recurrence** |  |  |  |
| Country: China vs Korea | -0.649 (-2.002, 0.703) | 0.425 | 0.224 |
| Mean age: ≥65 years vs <65 years | 0.575 (-0.193, 1.343) | 0.241 | 0.097 |
| Mean age (continuous) | 0.064 (-0.066, 0.195) | 0.041 | 0.216 |
| Sample size: ≥5000 vs <5000 | -0.509 (-1.086, 0.069) | 0.181 | 0.068 |
| Study time: ≥5 years vs <5 years | 0.649 (-0.703, 2.001) | 0.425 | 0.224 |
| TyG index condition: TyG index quartiles vs other | -0.649 (-2.001, 0.703) | 0.425 | 0.224 |
| High quality: Yes vs No | -0.210 (-1.546, 1.127) | 0.420 | 0.652 |
| **Poor functional outcome** |  |  |  |
| Country: China vs other | -0.423 (-2.207, 1.362) | 0.561 | 0.506 |
| Mean age: ≥65 years vs <65 years | 0.475 (-0.448, 1.397) | 0.290 | 0.200 |
| Mean age (continuous) | 0.183 (-0.012, 0.377) | 0.061 | 0.059 |
| Sample size: ≥5000 vs <5000 | -0.475 (-1.397, 0.448) | 0.290 | 0.200 |
| Study time: ≥5 years vs <5 years | 0.307 (-1.012, 1.626) | 0.414 | 0.513 |
| TyG index condition: TyG index quartiles vs other | -0.475 (-1.397, 0.448) | 0.290 | 0.200 |
| High quality: Yes vs No | -1.583 (-4.085, 0.918) | 0.786 | 0.137 |

Abbreviations: TyG, Triglyceride-Glucose.
