# Supplementary material for: The impact of triglyceride-glucose index on ischemic stroke: a systematic review and meta-analysis
Source: Cardiovasc Diabetol. 2023 Jan 6;22:2. doi: 10.1186/s12933-022-01732-0 (PMC9825038; doi:10.1186/s12933-022-01732-0)
Supplement: Supplementary file 7 — Additional file 7 Table S7. The publication bias of TyG index with prognosis among patients with ischemic stroke. [file 12933_2022_1732_MOESM7_ESM.docx]

**Additional file 7: Table S7. The publication bias of TyG index with prognosis among patients with ischemic stroke.**

| **Prognosis outcome** | **z** | **Begg's test** | **t** | **Egger's test** |
| --- | --- | --- | --- | --- |
| Mortality | 0.38 | 0.707 | 1.54 | 0.199 |
| Stroke recurrence | 1.71 | 0.086 | 4.05 | 0.027 |
| Poor functional outcome | 1.22 | 0.221 | 2.62 | 0.079 |
