# Supplementary material for: The impact of triglyceride-glucose index on ischemic stroke: a systematic review and meta-analysis
Source: Cardiovasc Diabetol. 2023 Jan 6;22:2. doi: 10.1186/s12933-022-01732-0 (PMC9825038; doi:10.1186/s12933-022-01732-0)
Supplement: Supplementary file 8 — Additional file 8: Fig S1. The publication bias assessment of the TyG index association with ischemic stroke risk. [file 12933_2022_1732_MOESM8_ESM.pdf]

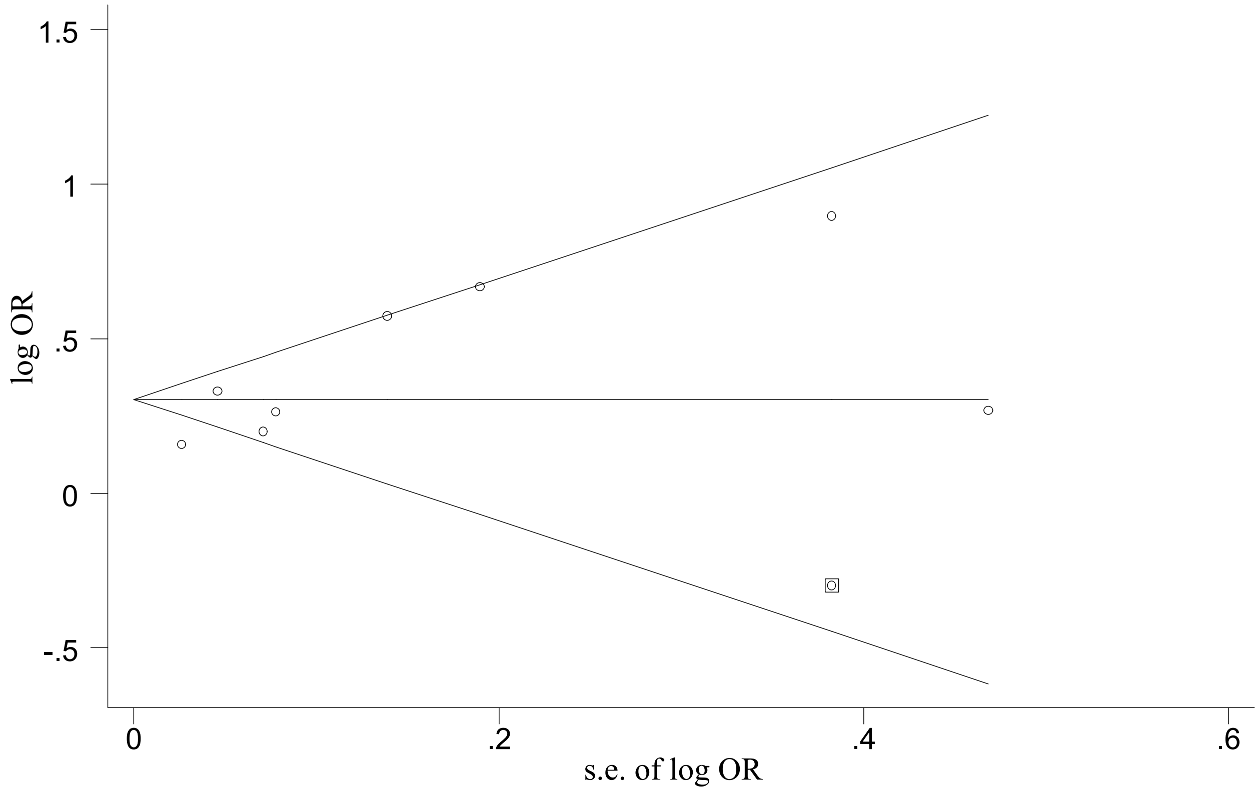

Additional file 8
Fig. S1

Additional file 8 Fig. S1 The publication bias assessment of the TyG index association with ischemic stroke risk.
