# Supplementary figures and images for: The impact of triglyceride-glucose index on ischemic stroke: a systematic review and meta-analysis
Source: Cardiovasc Diabetol. 2023 Jan 6;22:2. doi: 10.1186/s12933-022-01732-0 (PMC9825038; doi:10.1186/s12933-022-01732-0)

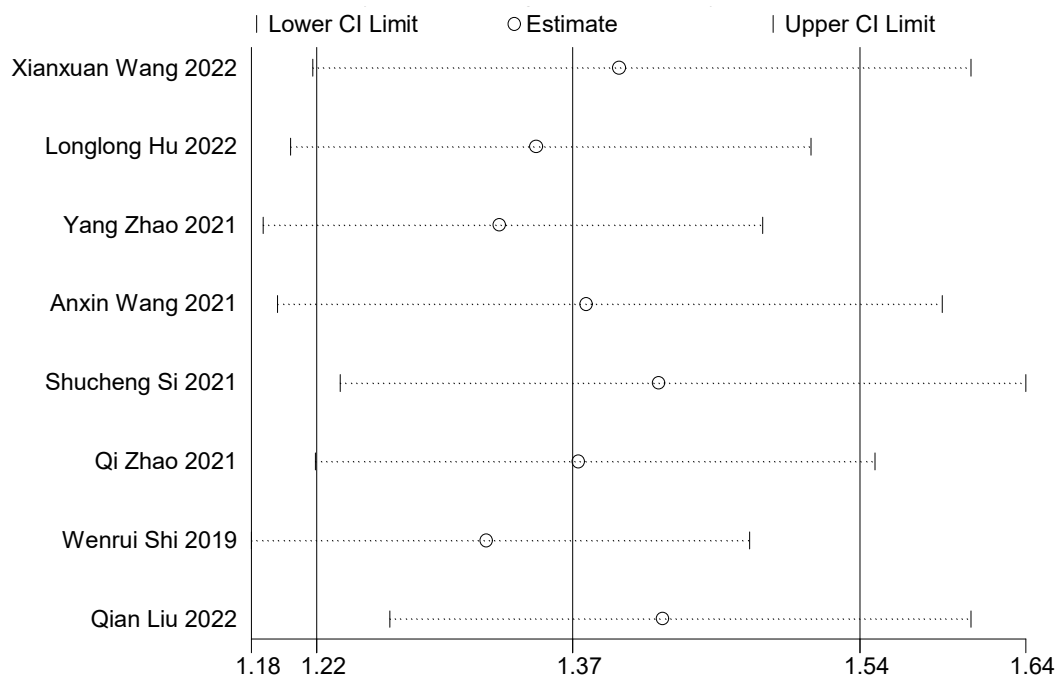

Additional file 9  
Fig. S2

Supplement: Supplementary file 9 — Additional file 9: Fig S2. The sensitivity analysis for the TyG index association with ischemic stroke risk. [file 12933_2022_1732_MOESM9_ESM.pdf]

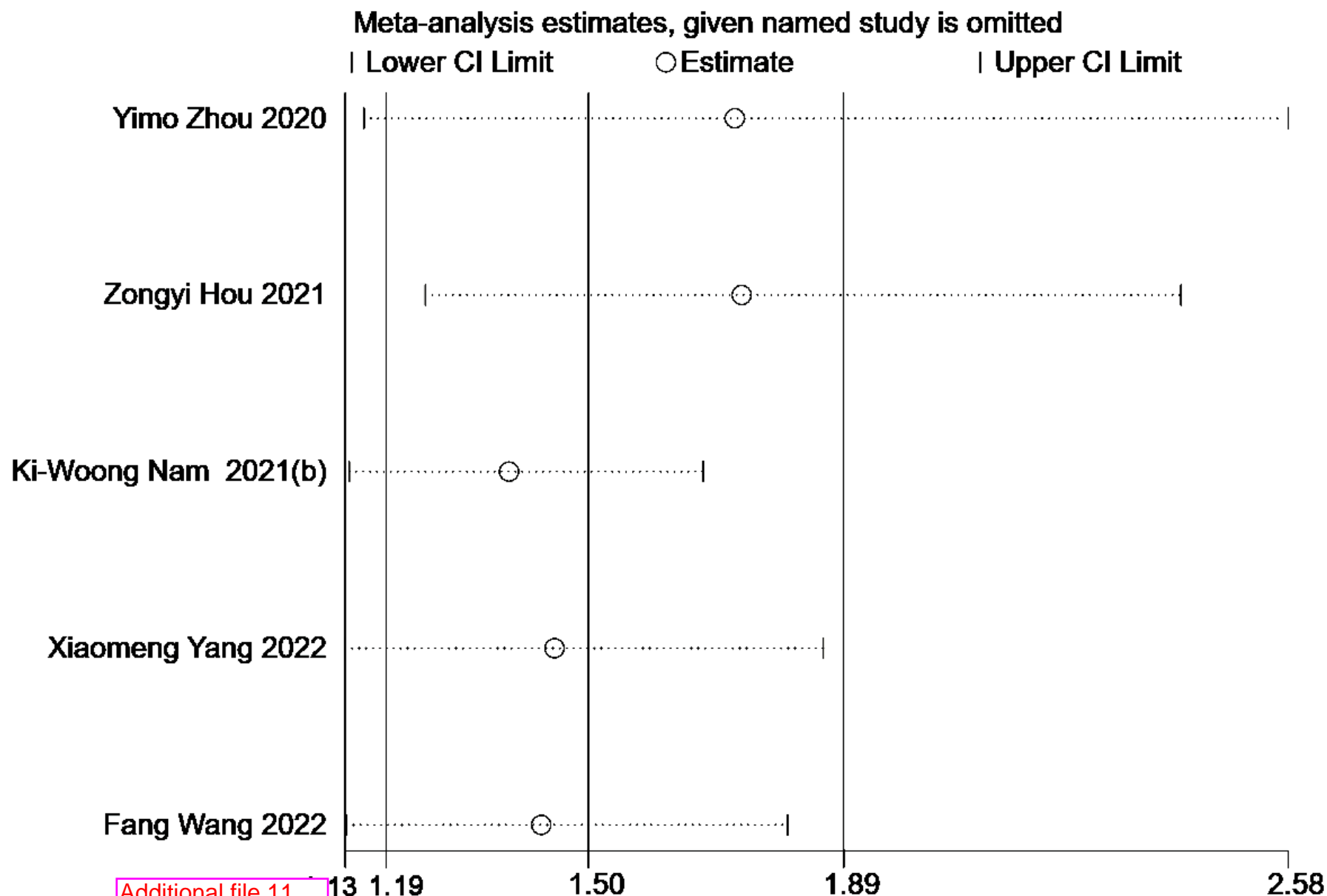

Additional file 11  
Fig. S4

Supplement: Supplementary file 11 — Additional file 11: Fig S4. Sensitivity analysis for the association of TyG index with Stroke recurrence among patients with ischemic stroke. [file 12933_2022_1732_MOESM11_ESM.pdf]

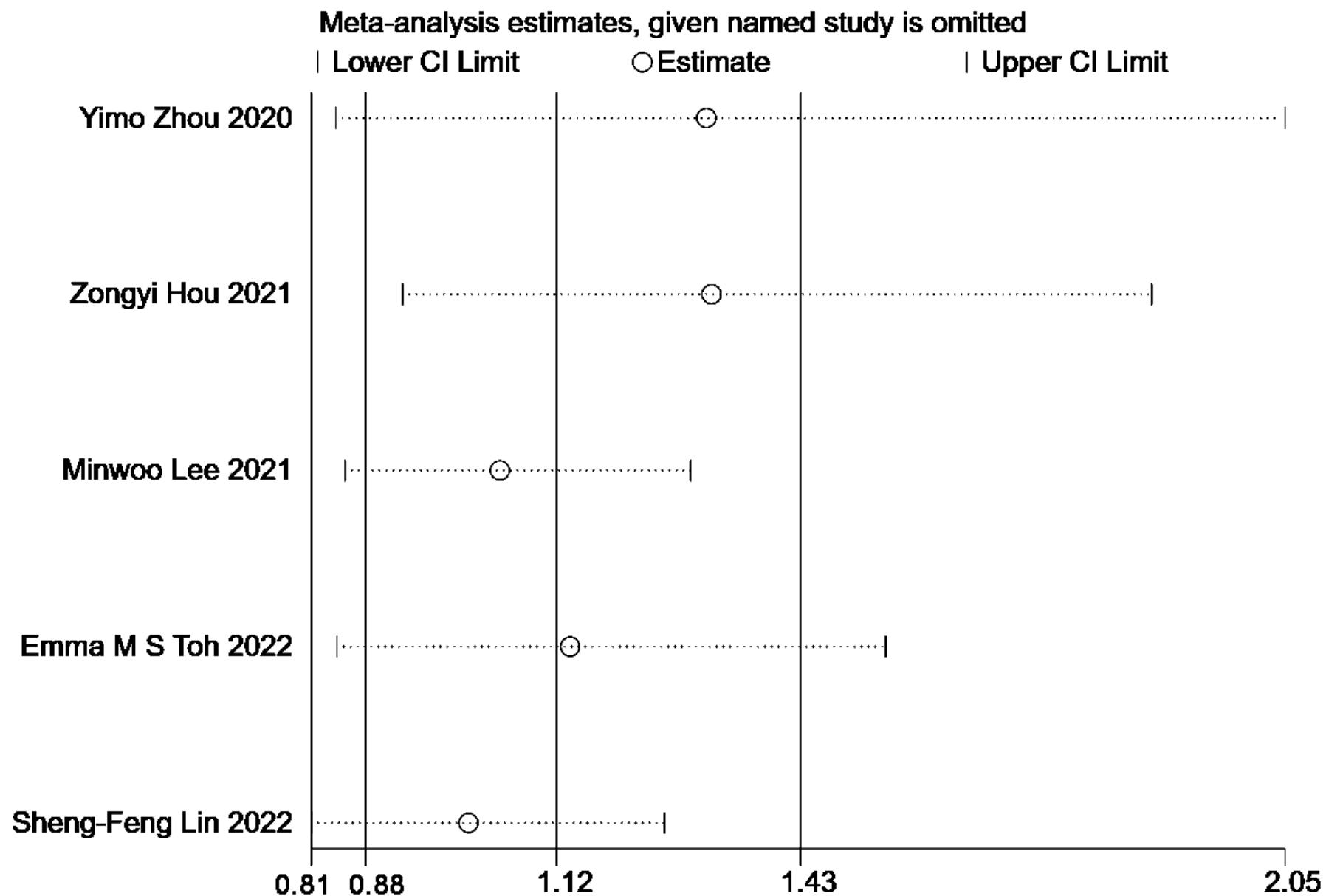

Supplement: Supplementary file 12 — Additional file 12: Fig S5. Sensitivity analysis for the association of TyG index with Poor functional outcome among patients with ischemic stroke. [file 12933_2022_1732_MOESM12_ESM.pdf]
