# Supplementary material for: The impact of triglyceride-glucose index on ischemic stroke: a systematic review and meta-analysis
Source: Cardiovasc Diabetol. 2023 Jan 6;22:2. doi: 10.1186/s12933-022-01732-0 (PMC9825038; doi:10.1186/s12933-022-01732-0)
Supplement: Supplementary file 10 — Additional file 10: Fig S3. Sensitivity analysis for the association of TyG index with Mortality among patients with ischemic stroke. [file 12933_2022_1732_MOESM10_ESM.pdf]

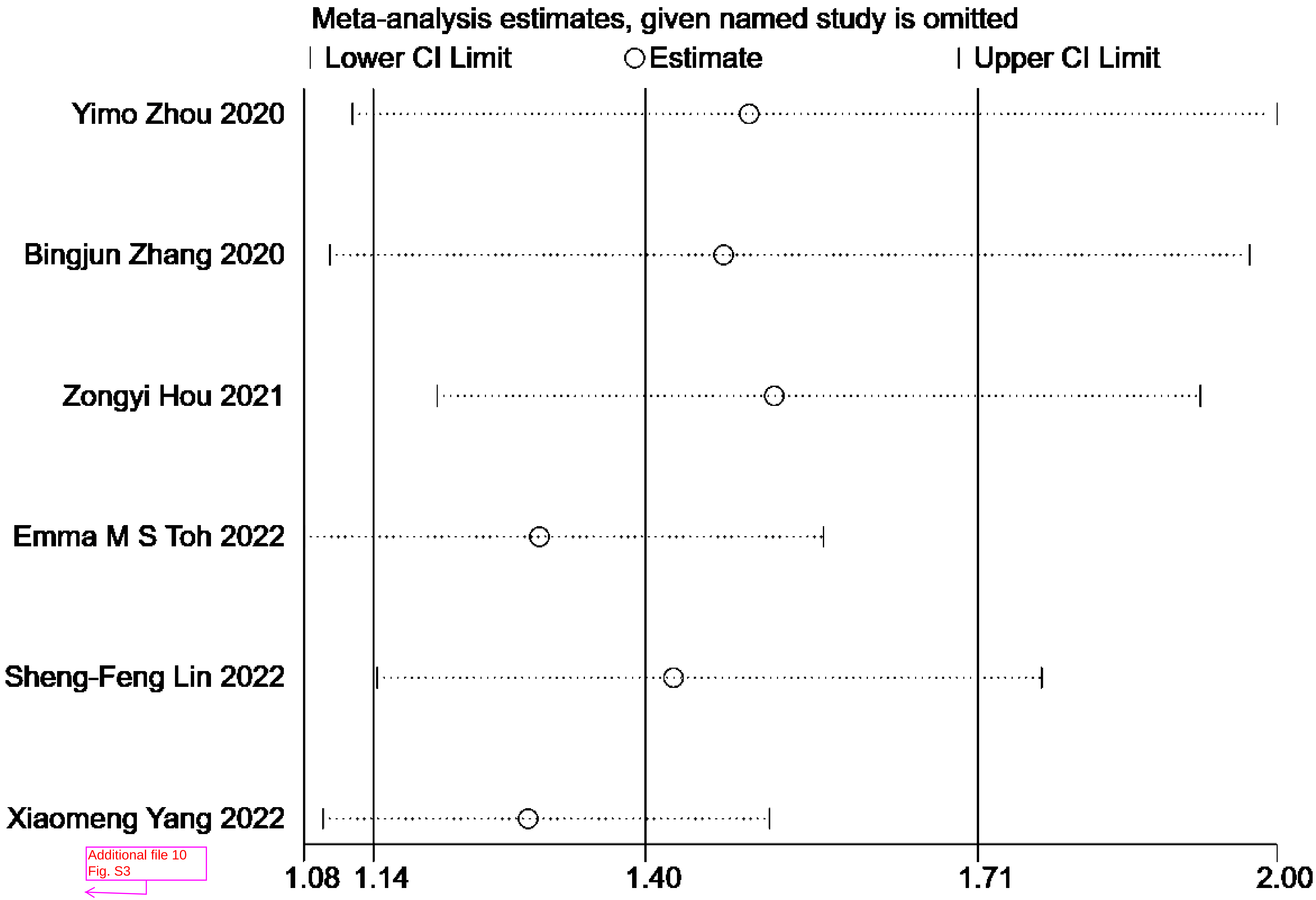

Additional file 10 Fig. S3 Sensitivity analysis for the association of TyG index with Mortality among patients with ischemic stroke.
